# Transcatheter mitral valve implantation in severe mitral annular calcification: a case report

**DOI:** 10.1093/ehjcr/ytae669

**Published:** 2024-12-31

**Authors:** Giulio Russo, Valerio Maffi, Gianluca Massaro, Gaetano Chiricolo, Giuseppe Massimo Sangiorgi, Aris Moschovitis, Maurizio Taramasso

**Affiliations:** Department of Biomedicine and Prevention, Cardiology Unit, Policlinico Tor Vergata, University of Rome, 00100 Rome, Italy; Department of Biomedicine and Prevention, Cardiology Unit, Policlinico Tor Vergata, University of Rome, 00100 Rome, Italy; Department of Biomedicine and Prevention, Cardiology Unit, Policlinico Tor Vergata, University of Rome, 00100 Rome, Italy; Department of Biomedicine and Prevention, Cardiology Unit, Policlinico Tor Vergata, University of Rome, 00100 Rome, Italy; Department of Biomedicine and Prevention, Cardiology Unit, Policlinico Tor Vergata, University of Rome, 00100 Rome, Italy; HerzZentrum Hirslanden, 8032 Zurich, Switzerland; HerzZentrum Hirslanden, 8032 Zurich, Switzerland

**Keywords:** Transcatheter mitral valve implantation, Mitral annular calcification, Valve-in-MAC, Mitral regurgitation, Mitral stenosis, Case report

## Abstract

**Background:**

Mitral annular calcification (MAC) is characterized by severe calcification of mitral annulus and might be associated with both mitral regurgitation and stenosis. It is technically challenging for both surgical and percutaneous approach and is burdened by high mortality.

**Case summary:**

The present case report describes a complex case of mitral steno-insufficiency (baseline transvalvular gradient = 5 mmHg, effective regurgitant orifice area 0.45 cm^2^, vena contracta 0.8 cm), due to MAC in an 83-year-old lady. In consideration of the clinical context (MAC) and patient’s several comorbidities and history of previous surgical interventions, she was deemed not suitable for surgery and a percutaneous treatment was selected (valve-in-MAC). Due to significant paravalvular leak, further implantation of a plug was required.

**Conclusion:**

The MAC represents a clinical and technical challenge for surgery. Transcatheter mitral valve implantation in MAC is a feasible alternative although it is technically challenging and burdened by high mortality. Detailed procedural planning is of utmost importance to achieve successful outcomes.

**Learning points**

- Mitral annular calcification (MAC) may be associated to both stenosis and regurgitation and represents a clinical and technical challenge for both surgical and percutaneous interventions.
- Transcatheter approach (valve-in-MAC) in technically feasible but requires careful planning.
- Left ventricle outflow tract obstruction, paravalvular leak, and prosthesis migration represent major concerns for valve-in-MAC.

## Introduction

Mitral annular calcification (MAC) is a degenerative condition characterized by progressive calcium deposition along the mitral annulus and represents a distinct entity from rheumatic mitral disease. It may cause both mitral valve stenosis (more frequently) and regurgitation. Its frequency may vary according to age, reaching up to 40% in elderly. Moreover, it may be found in almost half of the patient undergoing transcatheter aortic valve implantation.

In general, it is burdened by high mortality due to high-risk profile and several technical challenges. For these reasons, often patients affected by MAC are deemed unsuitable for surgery. Transcatheter mitral valve implantation (TMVI) in MAC (valve-in-MAC) has emerged an alternative option over the last years. We present a case of severe mitral regurgitation (MR) due to MAC treated by percutaneous implantation of mitral prosthesis.

## Summary figure

**Table:** 

2010	Chronic coronary syndrome treated with four coronary artery bypass
2019	Moderate MR combined with moderate aortic stenosis
2023	Worsening dyspnoea with severe MR at echocardiography
2024	TMVI in MAC

## Case presentation

**Figure 1:**
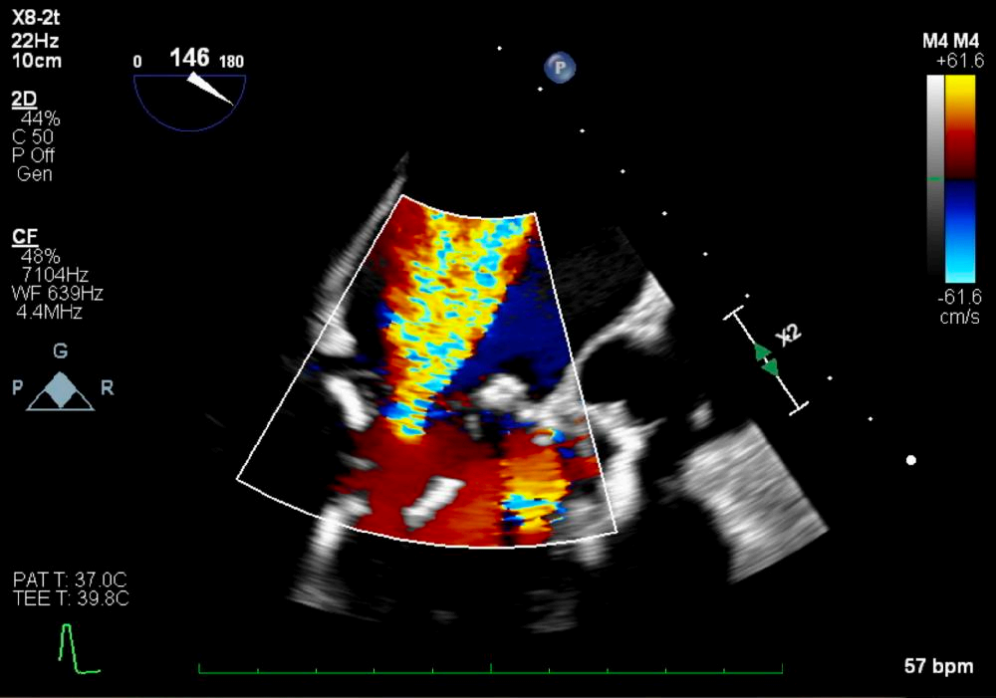
Mitral regurgitation at baseline as assessed by transesophageal echocardiogram.

**Figure 2:**
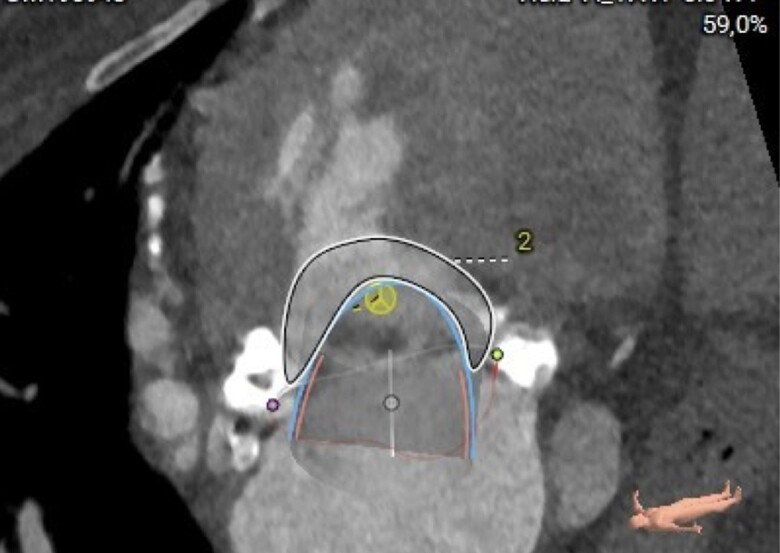
Procedural planning with computed tomography scan and assessment of neo-left ventricle outflow tract of 329 mm^2^ (indicated by the area with number 2).

**Figure 3:**
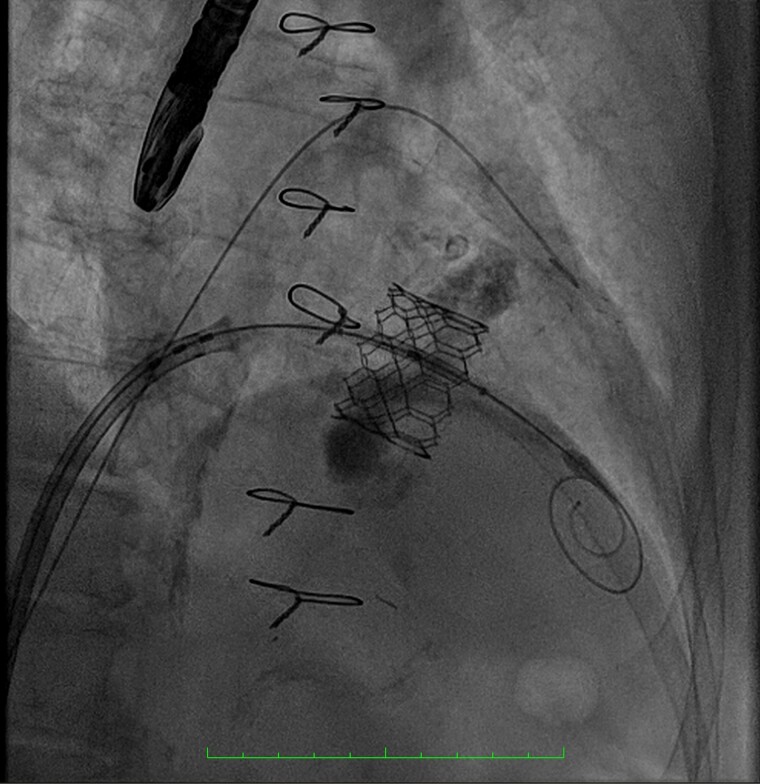
Implantation of prostheses in mitral position.

**Figure 4:**
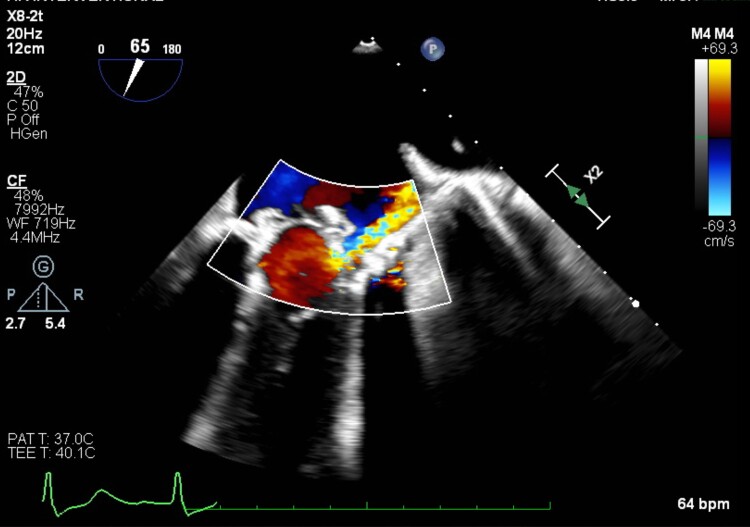
Residual paravalvular leak in 2D echocardiogram.

**Figure 5:**
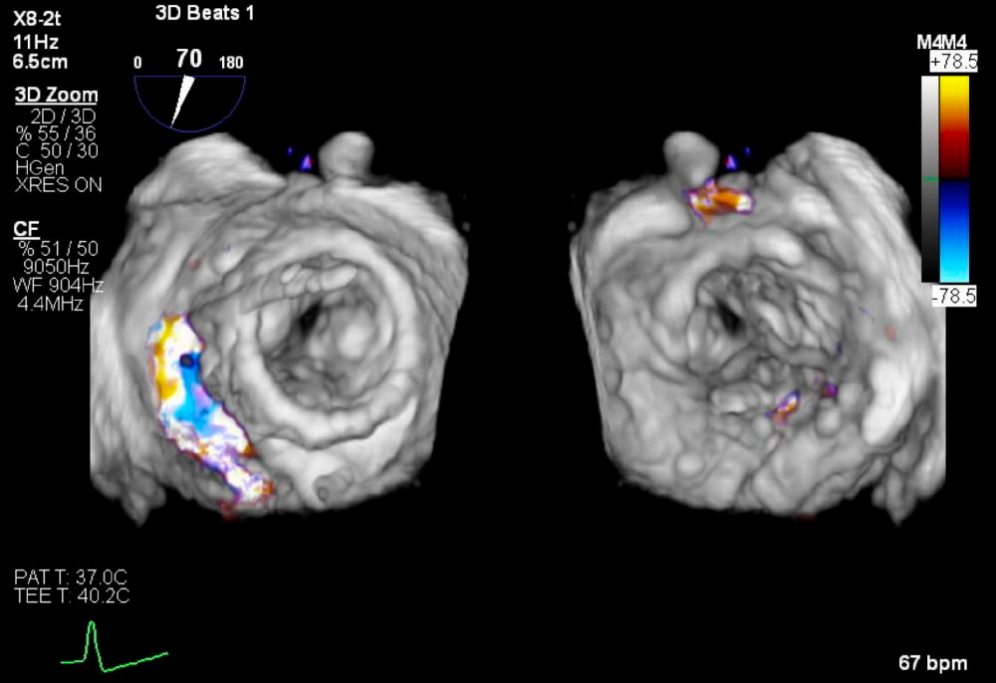
Residual paravalvular leak in 3D echocardiogram.

**Figure 6:**
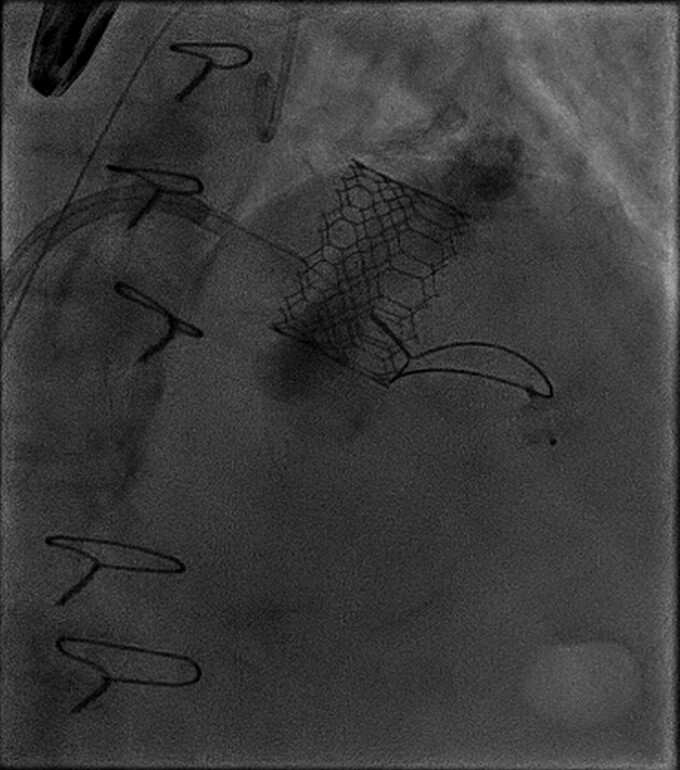
Plug snaring.

**Figure 7:**
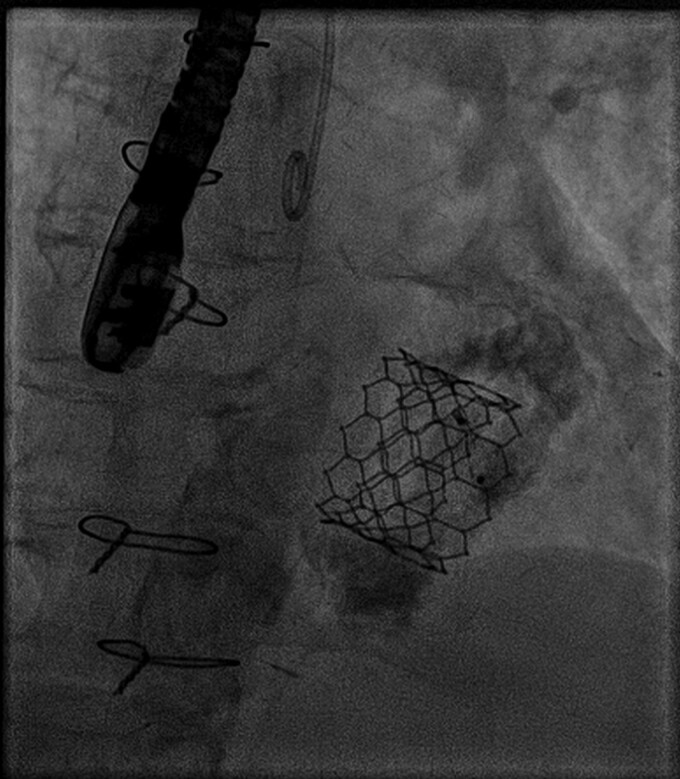
Plug implantation.

An 83-year-old lady was referred to our centre because of worsening dyspnoea (New York Heart Association, NYHA III–IV). The patient had all cardiovascular risk factors (hypertension, diabetes, smoking, dyslipidaemia, and positive familiar history for cardiovascular disease) and persistent atrial fibrillation under anticoagulation with vitamin K antagonists and had previously undergone three coronary artery bypass graft (CABG). At physical examination, a systo-diastolic murmur was present at the apex. The echocardiogram showed good left ventricular function (left ventricular ejection fraction, LVEF = 60%) with mild-to-moderate aortic stenosis, severe mitral steno-insufficiency (transvalvular mean gradient = 5 mmHg, effective regurgitant orifice area 0.45 cm^2^, vena contracta 0.8 cm) with severe MAC, and severe tricuspid regurgitation (*Figure 1*).^1^ No CABG disease was reported at angiography. The clinical was discussed at local heart team and deemed high risk for surgery (STS score > 8, EuroScore II 29.47%) and unsuitable for transcatheter mitral edge-to-edge repair.^2,3^ Consequently, the patient was screened for transvenous–transseptal percutaneous valve-in-MAC with computed tomography (CT) scan and deemed suitable for the procedure (*Figure 2*): in particular, calcium burden and distribution were assessed with a total MAC score of 9 points together with neo-LVOT simulation.^4^ After screening failure for Tendyne prosthesis (Abbott, USA), Sapien 3 Ultra was selected (Edwards Lifesciences, USA). After transseptal puncture in the posterior–inferior quadrant of the fossa ovalis, the patient underwent successful TMVI with Sapien 3 Ultra 29 mm (Edwards Lifesciences, USA)^5,6^ (*Figure 3*). In addition, due to significant residual paravalvular leak (PVL), a plug (Amplatzer Valvular Plug III, AVP III, Abbott, USA) was implanted (*Figures 4* and *5*). The first attempt was unsuccessful and the AVP III embolized requiring recapture with the snare (*Figure 6*). It was finally successfully implanted at second attempt (*Figure 7*). Post-procedural echocardiogram showed no residual MR (transvalvular gradient 1 mmHg) and good LVEF (see Supplementary material online, *Video S1*).

The patient was discharged 7 days after the procedure in good clinical status, and at 30-day follow-up, the patient was alive.

## Discussion

Currently, MAC represents the most complex subtype of mitral valve disease and is burdened by high mortality for both surgery and transcatheter therapies due to high-risk profile of patients and to technical anatomic challenges.^7^ It may be associated to both mitral stenosis and MR: in the first case, reduced annular dilatation during diastole and restriction of the anterior mitral leaflet motion may represent the main mechanism responsible for the stenosis, while in the second case, the combination of several factors leads to MR (e.g. the failure of the calcified annulus to contract at the end of diastole and calcium infiltration of the base of the leaflets reducing their mobility, increasing traction on the chordae, and elevating the leaflets).

While European guidelines indicate surgery as the first option therapy for most cases of mitral valve disease, MAC is often deemed unsuitable for surgery due to its intrinsic high operative risk.^8^ Consequently, the recent development of percutaneous technologies gradually leads to consider transcatheter therapies as a possible alternative for MAC.^9^ So far, results have been contrasting demonstrating the technical feasibility in some selected cases but confirming high mortality also for percutaneous approach. Indeed, according to a recent registry, 30-day and 1-year mortality may reach 35% and 63%, respectively.^6,10^ Such outcomes may be explained by several issues:

- Left ventricular outflow tract obstruction (LVOTO): It is due to the implanted prosthesis pushing the anterior mitral leaflet towards the septum. It represents the most important concern for TMVI as it may occur in up to 40% in valve-in-MAC predicting high 1-year mortality rate, and at the same time, it is the most frequent cause of screening failure. Several percutaneous techniques have been investigated over the last years in order to reduce the risk of LVOTO including intentional anterior mitral leaflet laceration, alcohol septal ablation, and, for patients with previous edge-to-edge intervention, the so-called ElastaClip.^11^
- Prostheses fixation: MV anatomy is characterized by asymmetrical shape of the annulus and leaflets, large annular dimensions, absence of calcifications in most cases, and a complex subvalvular anatomy. Due to the complex anatomy, sealing and reliable fixation represent another important concern of TMVI. Radial forces as the only mechanism providing fixation might be not enough and might increase the risk of compression and damage to adjacent structures such as the LVOT, the conduction system, the coronary sinus, the left circumflex artery, and the aortic root. Moreover, the dynamic, D-shaped of native MV may affect both fixation and sealing contributing to prostheses embolization and to PVL.
- Durability and thrombogenicity: Currently, no data exist about the long-term durability of transcatheter prostheses in mitral position. According to surgical experience with bioprosthesis, the degeneration process begins 5 years after MV intervention and the freedom from structural valve deterioration varies from 70% to 90% at 10 years. Multiple factors influence the thrombogenicity and degeneration risk. Most of them are valve-related factors and include device profile, shape, and leaflet/stent material. Based on the experience gained so far, although no univocal approach in terms of drugs choice and duration of therapy has been established, it is highly advisable to use at least one antiplatelet/anticoagulant drug, with anticoagulant being preferred over antiplatelet.

The case presented shows all the complexities of patients affected by MAC. In particular, the patient was excluded from surgery because of age, prior cardiac surgery, and multiple comorbidities with high/prohibitive risk according to both STS score and EuroScore II. The patient was therefore selected for percutaneous valve-in-MAC after adequate screening with CT scan. According to calcium distribution and valve anatomy, the risk of PVL and consequent need for a plug implantation was considered already before the procedure. Indeed, despite successful prosthesis implantation, as a consequence of high calcific annulus, the patient required PVL closure.

## Conclusions

Transcatheter mitral valve-in-MAC represents a feasible procedure although it remains a highly challenging scenario. Heart team and appropriate procedural planning play a key role to achieve optimal outcomes.

## Supplementary Material

ytae669_Supplementary_Data

## Data Availability

Data are available upon reasonable request.

## Lead author biography

Giulio Russo is an interventional cardiologist at Policlinico Tor Vergata University Hospital in Rome with specific interest in complex coronary interventions and structural heart disease interventions.

## Supplementary material

**Consent:** The patient gave consent to undergo the procedure and to publish the clinical case. I confirm to have respected the COPE guidelines.

**Funding:** None declared.
